# Exploring the Prevalence of Disrespect and Abuse during Childbirth in Kenya

**DOI:** 10.1371/journal.pone.0123606

**Published:** 2015-04-17

**Authors:** Timothy Abuya, Charlotte E. Warren, Nora Miller, Rebecca Njuki, Charity Ndwiga, Alice Maranga, Faith Mbehero, Anne Njeru, Ben Bellows

**Affiliations:** 1 Population Council, P.O. Box 17643–00500, Nairobi, Kenya; 2 Population Council, 4301 Connecticut Ave, NW #280, Washington, District of Columbia, 20008, United States of America; 3 Woman Care Global, 12400 High Bluff Drive, Suite 600, San Diego, California, 92130, United States of America; 4 Centre for Population Health Research and Management, P.O Box 19607–00202, Nairobi, Kenya; 5 Federation of Women Lawyers, P.O. Box 46324–00100, Nairobi, Kenya; 6 National Nurses Association of Kenya, 49422–00100, Nairobi, Kenya; 7 Division of Reproductive Health, Ministry of Health, P. O. Box 43319–00100, Nairobi, Kenya; Johns Hopkins Bloomberg School of Public Health, UNITED STATES

## Abstract

**Background:**

Poor quality of care including fear of disrespect and abuse (D&A) perpetuated by health workers influences women’s decisions to seek maternity care. Key manifestations of D&A include: physical abuse, non-consented care, non-confidential care, non-dignified care, discrimination, abandonment, and detention in facilities. This paper describes manifestations of D&A experienced in Kenya and measures their prevalence.

**Methods:**

This paper is based on baseline data collected during a before-and-after study designed to measure the effect of a package of interventions to reduce the prevalence of D&A experienced by women during labor and delivery in thirteen Kenyan health facilities. Data were collected through an exit survey of 641 women discharged from postnatal wards. We present percentages of D&A manifestations and odds ratios of its relationship with demographic characteristics using a multivariate fixed effects logistic regression model.

**Results:**

Twenty percent of women reported any form of D&A. Manifestations of D&A includes: non-confidential care (8.5%), non-dignified care (18%), neglect or abandonment (14.3%), Non-consensual care (4.3%) physical abuse (4.2%) and, detainment for non-payment of fees (8.1). Women aged 20-29 years were less likely to experience non-confidential care compared to those under 19; OR: [0.6 95% CI (0.36, 0.90); p=0.017]. Clients with no companion during delivery were less likely to experience inappropriate demands for payment; OR: [0.49 (0.26, 0.95); p=0.037]; while women with higher parities were three times more likely to be detained for lack of payment and five times more likely to be bribed compared to those experiencing there first birth.

**Conclusion:**

One out of five women experienced feeling humiliated during labor and delivery. Six categories of D&A during childbirth in Kenya were reported. Understanding the prevalence of D&A is critical in developing interventions at national, health facility and community levels to address the factors and drivers that influence D&A in facilities and to encourage clients’ future facility utilization.

## Background

Multiple factors are impeding progress in attaining the fifth Millennium Development Goal of reducing maternal mortality and increasing universal access to reproductive health. These include inequities in financial and geographic access to quality services, health worker distribution, and weak management capacities, which limit reproductive health service demand. In addition, high dual chronic and infectious disease burdens in populations at epidemiological transition along with patriarchal societies that dis-empower women contribute to stagnating or deteriorating reproductive health services. Low skilled birth attendance coverage, a key Millennium Development Goal 5 indicator [1], is associated with a high maternal mortality ratio, which, in many low-income settings, is estimated as one hundred times greater than in high-income countries [2]. One key strategy for addressing high maternal and newborn morbidity and mortality is to increase the proportion of women utilizing skilled care at birth. Progress has been slow for achieving the skilled birth attendance targets because improvements require overcoming cultural, financial, and geographic barriers to its access, as well as reforming poor quality of care at facilities [2, 3].

An important but little understood component of poor care that women receive during childbirth in facilities is disrespect and abuse (D&A) perpetuated by health workers and other facility staff [4]. Fear of experiencing D&A negatively influences women’s decisions to seek care at a health facility during labor and delivery [5]. In Kenya, the skilled birth attendance rate fell from 50% in 1989 to 44% in 2008/9 [6], a likely contributor to the country’s sustained high maternal mortality ratio, which is currently 488 deaths per 100,000 live births [7]. The reasons for the low levels of skilled care at birth are relatively well-understood in Kenya [8]. In 2007, a report by the Federation of Women Lawyers and the Centre for Reproductive Rights, documented D&A during childbirth including physical abuse (pinching on thighs, slapping and beating), non-consensual care (coerced cesarean sections), non-dignified care, verbal abuse, discrimination towards poor and young mothers, abandonment of women during and after labor, and detention in facilities because of inability to pay [9]. In another study, Family Care International found that women did not attend facilities for fear of being insulted, assaulted, or abandoned [10]. Moreover, in the most recent Kenya Service Provision Assessment in 2010, women described doctors treating patients rudely (‘abused them’), ignoring them, drunk at work, or failing to fulfill their requisite hours of service. In the Kenya Service Provision Assessment, patient abuse was most commonly documented during labor in maternity units, where nurses occasionally shout at women or slap them [11].

Despite these observations, the extent of D&A during facility-based deliveries has not been systematically documented or well defined [12]. Identifying both aggravating and mitigating factors of negative and abusive provider-patient relationships has been neglected in health systems research, especially during childbirth [4]. D&A in childbirth is a critical but less discussed barrier to skilled birth attendance utilization, which constitutes a common cause of suffering and a human rights violation for women in many countries [4, 12]. Poor provider attitudes and poor relationships with clients are an important barrier to health care, yet efforts to measure and institutionalize interventions to improve these relationships are limited. Abundant evidence exists on improving technical quality of care but efforts targeting the often difficult to measure and document “*soft issues*” of provider-client relationships are limited. One pertinent “*soft issue*” not well discussed is the extent to which D&A occurs when clients seek care, with less evidence on its extent during labor and delivery, which can be described as “a *vulnerable moment*” during the birthing process.

A landscape analysis by Bowser and Hill reviewed evidence of D&A in facility deliveries to define the concept, identify its scope, contributing factors, and impact in childbirth, along with potential interventions [12]. Based on their review, D&A was categorized into seven manifestations: physical abuse, non-consensual care, non-confidential care, non-dignified care, discrimination, abandonment of care, and detention in facilities. Key contributing factors for these behaviors are grouped as individual and community level factors normalizing D&A, lack of legal and ethical foundations for addressing D&A, lack of leadership, standards and accountability, and provider prejudice due to lack of training and resources [12].

Despite Bowser and Hill’s description of the D&A categories, there is limited evidence about the extent to which the categories manifest in developing country settings, what are the measurable D&A elements, and their prevalence. This paper describes a study that seeks to contextually define the types of D&A behaviors that manifest in selected facilities in Kenya and to measure their prevalence.

## Methods

### Developing measurable construct of D&A

To translate the categories of D&A identified in the review [12] into measurable domains, investigators from two USAID-TRAction funded projects (in Kenya and Tanzania) met to harmonize and contextualize the working definitions of D&A during childbirth. The team discussed research methodologies and developed common definitions of D&A in a Construct Map. A detailed description of the definitions is published separately, focusing on normative and experiential building blocks [13]. The focus of the current measurement is based on experiential building block that took account of women’s experiences of disrespect and abuse. These were a specific set of behaviors or conditions agreed by all stakeholders to constitute disrespect and abuse. The basis of this definition is that if the goal is to promote women’s dignity in childbirth, then it matters if a woman experiences her treatment as disrespectful and abusive. Such an experience is likely to influence future decisions about where to deliver and whether to recommend that facility to others [13].

The second dimension of definition of D&A includes the normative building block which comprise codes of behavior or infrastructural standards, where departure from these standards could be considered violations constituting D&A. The normative block has four key dimensions: human rights law, domestic law, ethical codes and local consensus on behaviors [13].

The experiential building block, refers to events or conditions considered as D&A, regardless of patient experience or provider intention and classified into three dimensions: 1) subjective experiences whereby women experience D&A even if it does not result from actions observed; 2) objective events or conditions that are observable actions experienced or intended as such; and 3) intentionality, whereby a woman does not interpret an action as D&A, but the provider actually intends it as disrespectful or abusive [13]. Subjective experience of D&A was measured through the client exit survey described in this paper. Table 1 outlines the normative and evidentiary building blocks and provides examples of actions and behaviors that may be experienced as disrespectful and how they link to the building blocks.

**Table 1:** Normative and evidential building blocks: the link between definitions of disrespect and abuse, and the list of actions and behaviours used in measurement.

Normative Building block	Definition	Category of D&A ^a^	Evidentiary Building Block: Examples of actions or behaviors that are reported	Questions asked
Human rights law	Right to health, freedom from abusive behavior and entitlement to facility conditions that are, accessible, affordable, acceptable and of good quality (AAAQ).	Physical abuse	Pinching/slapping/pushing/beating/ poking Rape/ sexual harassment.	At any point during your stay for this delivery were you physically abused by any of the healthcare workers? For example physical abuse might include being hit or slapped.
		Detention	Detained when a woman is unable to pay for services.	At any point during your stay for this delivery were you or your baby prevented from leaving this facility because you could not pay?
		Non confidential	HIV status shown to others; health information discussed with non-health staff; uncovered during delivery or examination; no screens blocking view during delivery or examination; discussed her issues when other clients were listening	At any point during your stay for this delivery were you treated in a way that violated your privacy? At any point during your stay for this delivery were you treated in a way that violated your confidentiality?
Domestic law	Malpractice or criminal wrongs such as assault.	Corruption	Request for a bribe for services.	At any point during this delivery in this facility did you feel/ perceive or were you asked by anyone for money other than the official cost of service to access services or any favors.
Ethical codes	Standards of conduct for members of medical /nursing professions and national standards of care developed by the MOH.	Non consented care	No permission obtained before examination for medical procedures such as tubal ligation, hysterectomy.	At any point during your stay for this delivery was any treatment done to you without your permission?
		Abandonment /neglect	Ignored when sought help for pain relief or left unattended by heath workers when they needed help.	At any point during your stay for this delivery were you left un attended by health providers when you needed care?
Local consensus	Specific set of behaviors or conditions that patients, families, providers, agree constitute inexcusable D&A.	Non dignified care	Use of non-dignified language such as shouting and scolding; Threats of withholding services /threatened with going to theatre, called insulting names, laughed or scorned at	At any point during your stay for this delivery did any healthcare provider talk to you or use a tone or facial expression that made you feel uncomfortable?

Note: Normative building blocks comprise codes of behavior or infrastructural standards, and departure from these standards could be considered violations constituting D&A. The normative block has four key dimensions: human rights law, domestic law, ethical codes and local consensus on behaviors The evidentiary building block, meanwhile, refers to events or conditions considered as D&A, regardless of patient experience or provider intention, classified into three dimensions: 1) subjective experiences whereby women experience D&A even if it does not result from actions observed; 2) objective events or conditions that are observable actions experienced or intended as such;; and 3) intentionality, whereby a woman does not interpret an action as D&A, but the provider actually intends it as disrespectful or abusive.

With a set of definitions, measurement instruments were developed and validated through qualitative interviews with clients to identify potential gaps in the Construct Map. A client exit tool was developed and validated through an exit survey conducted among 75 respondents. In order to check the reliability of the exit tool in estimating the prevalence of D&A, we further conducted follow-up case narratives two weeks later among 25 participants who reported any form of D&A in the exit survey and 25 others how did not report any form of D&A. The outcome of this analysis enabled us to refine the tools for measuring the prevalence of D&A.

## Study Design

This paper is based on cross sectional analysis of baseline data from a quasi-experimental study designed as a before-and-after without a comparison to measure the effect of interventions in reducing the prevalence of D&A experienced by women during labor and delivery in health facilities in Kenya. Initially, the study was designed as a before and after design with the facilities and populations identified as intervention sites and equivalent comparison facilities and population living around health facilities not served by the D&A program in order to control for potential time dependent confounders [14]. However due to political challenges in selecting intervention and comparison facilities, a before-and after implementation research study without comparison was adopted. All facility and populations around facilities were included in the intervention through a step wise implementation over a period of one year. This study is embedded in an ongoing Population Councils' reproductive health vouchers evaluation project supported by the Bill and Melinda Gates Foundation [15]. The data collection was conducted between September 2011 and February 2012.

## Study Sites

Thirteen facilities included in the voucher project evaluation were purposively selected in Kisumu, Kiambu, Nyandarua and Uasin Gishu sub counties, along with one maternity hospital in Nairobi. The three facilities from each sub county that were selected represented different facility types (public, private and faith based) and different levels of care (hospitals, nursing homes, health centers and referral facilities) and were relatively similar in number of deliveries, professional expertise, skills distribution, clientele, location and fees charged, among others. Study facilities had a total of 58 specialist doctors, 116 medical doctors, and 1503 nurses or midwives, 27 theater nurses, 48 anesthetists and 126 pharmacists with variations by level of care. The bed capacity for labor wards was 135 and 42 in the delivery rooms.

## Study Procedures

Exit interviews with women discharged from postnatal wards measured experienced D&A within the evidentiary building block. Due to the sensitivity of the issues raised, prior to any data collection officers from the Division of Reproductive Health (DRH) of the national Ministry of Health and the study coordinator visited each selected district to provide information to the facility management and staff about the study. This was done two weeks prior to the study activities. Research assistants were trained to conduct the exit interviews in five-day training with a broad introduction to the research objectives, observational skills, and ethical issues. In addition, Research assistants were provided with information referring clients requiring additional support.

The sample size calculation was based on the larger before-and-after study that aimed to measure the effect of the intervention package on the primary outcome indicator “reduction in the prevalence of D&A in facilities”. Due to lack of a previous measure for D&A in the literature, the study utilized an estimated 22.2% of women who reported not using facilities due to provider related reasons in the voucher evaluation survey conducted in 2010 around the same facilities [15]. The assumptions were that provider related reasons were associated with humiliating behavior or perceived to be disrespectful by the clients. This was used as baseline measure for the interventions. The study was thus designed to measure a 10% decrease of D&A, with 90% estimated power for one-sample comparison of proportion with two sided alpha of 0.005 and an estimated design effect of 2 to account for facility clustering, resulting in a sample size of 583, with a 10 percent over sampling providing a total sample size of 641. To increase the robustness of the study, the final sample size calculation was powered at 90% up from the 80% initially proposed in the protocol [14). Data for this paper is therefore drawn from a cross sectional baseline survey conducted as part of the implementation research designed as a before-and-after design without a comparison to measure the effect of interventions in reducing occurrence of D&A.

### Client exit interviews

The exit survey sampled women of reproductive age, between 15 to 45 years, who received maternity services from the 13 study facilities [14]. To capture D&A prevalence for subjective experience, client exit interviews were conducted with women who had just given birth. Interviews were conducted once women had been discharged from the postnatal ward but within the hospital compound in a private place. The questionnaire was developed through a series of discussions with the research teams from Kenya and Tanzania. Focus group discussions with women and men also helped determine D&A taxonomy, with the tools pre-tested within the local context, and re-tested. The questionnaire comprises several modules: demographics, household characteristics including socio-economic status, past service utilization, delivery characteristics, perceived quality and satisfaction, and D&A experience. The primary question of assessing the overall prevalence of D&A was whether the woman was treated in a way that made her feel humiliated or disrespected during all the labor and childbirth experience. The questions used for each category of D&A are presented in the last column in Table 1.

To implement the study, researchers approached all postnatal women both recently delivered and discharged from the postnatal ward, describing the nature of the study and interview process, emphasizing its privacy and confidentiality. Mothers of newborn babies who were physically detained in the facility for non-payment or clearance of bills associated with the current birth were also included. All women satisfying the inclusion criteria were recruited until the required sample size were reached.

### Data management and analysis

Portable Digital Assistants (PDAs) were used to record the exit interviews. PDA data were downloaded into a Microsoft Access database prior to Stata 11 analysis. Tests of proportions and relationships between key variables were at 1% and 5% level of significance. Descriptive statistics were computed using the chi square test for categorical variables. Frequencies and percentages of different D&A manifestations are reported in the accompanying table. The key outcome variables for self-reported D&A (subjective) include physical abuse, non-dignified care, non-confidential care, non-consensual care, abandonment, detention, and corruption. A multivariate fixed effects logistic regression model that accounted for facility clustering examined the relationship between D&A and demographic factors. Results are presented as adjusted odds ratios (OR). Throughout the analysis, we identified patterns of missing data and their distribution. For cases where missing data was as result of skip patterns or non-response, only data available for each variable were analyzed.

The basic model for reported D&A is given by (Eq 1) where π_*ij*_ is the probability of experiencing the outcome for individual *i* identified from a facility *j*; *X*_*ij*_ is the vector of covariates; *β* is the associated vector of fixed parameters; and *μ*_*j*_ are the unobserved characteristics of client experiences that might be correlated with the outcomes.

$$\text{log}it(\pi_{ij})\;=\;X_{ij}\beta +\mu_{j}$$ (1)

The independent variables of interest for reported D&A included age, marital status (either currently married or never married/other), education, parity, service satisfaction, time of delivery, past experience of physical or sexual abuse, history of depression, the presence of a support person during childbirth, and socioeconomic status (SES). SES was calculated using principal components analysis to create income quintiles from household assets then dichotomized into two categories (lowest 20% and highest 80% of wealth quintiles).

### Ethical issues

Women were asked a number of sensitive questions including reproductive behavior and aspects of D&A. Therefore, careful steps during the questionnaire design were aimed to minimize potential informant discomfort. Study tools were pre-tested among a small group of women with characteristics similar to the study population to identify potentially negative consequences, and were modified accordingly. To avoid the risk of others overhearing informants’ information, interviews were conducted in private settings, with ample time for data collection to guarantee privacy and confidentiality. Provisions were made to train researchers to ensure that guidance on ethical conduct is clearly understood and implemented. The research team was trained to listen and observe intently without displaying any judgmental attitude about information from informants and on other critical ethical issues on gathering information from women.

All interviews followed participants’ written informed consent. From the outset, participants were clearly informed that they had a right to withdraw at any time. Before both the interview and any consent for their participation was sought, participants were provided with information about the study including its aim and methods, institutional affiliations, anticipated benefits and potential risks, potential discomfort including sensitive questions about sexual behavior (which they could choose not to answer), their right to abstain from participating or to withdraw at any time without reprisal, measures ensuring information confidentiality, contact details for the study coordinator for any questions or concerns, and the fact that monetary compensation was provided only if a participant had to travel for the interview. All of this information was read to potential participants, and once they understood and accepted, signed the informed consent form. All informed consent forms and questionnaires were translated into the relevant languages.

The research protocol was approved by the Division of Reproductive Health, Ministry of Health, the Kenya Medical Research Institute (KEMRI) Ethical Review Board (approval SCC No 288), and the Population Council’s Institutional Review Board (PC IRB 577). All informed consents forms used in this study were reviewed by both review boards. The boards were aware that potential participants may be under the age of 18 and would be providing consent for themselves.

## Results

### Characteristics of clients interviewed

Table 2 shows the characteristics of 641 postnatal women who were interviewed in the 13 facilities, with a mean age of 25 years. Majority of women 82% (n = 525), were married, 53% (n = 335) had completed secondary school or higher, and 58.6% (n = 374) were multiparous. More than half of the women interviewed reported feeling sad or depressed during the previous 12 months; a third had ever been ‘emotionally abused’; just under one fifth had ever been physically abused; and 2% reported ever being raped.

**Table 2:** Socio demographics and delivery experience characteristics of survey respondents from 13 facilities in Kenya.

Characteristics	
**Age of clients interviewed**	**n = 641(%)**
Average age (SD)	24.9 (5.3)
15–19 years	85 (13.2)
20–24 years	255 (39.8)
25–29 years	180 (28.1)
30–34 years	83 (12.9)
35–39 years	27 (4.2)
Above 40 years	10 (1.6)
**Marital status**	**n = 641 (%)**
Married /cohabiting	525 (81.9)
Never married	99 (15.4)
Separated /divorced	17 (2.7)
**Level of education attainment**	n = 631* (%)
Primary	296 (46.9)
Secondary	272 (43.1)
Tertiary	63 (9.9)
**Mother's parity**	**n = 641 (%)**
First birth	263 (41.3)
1–3 children	339 (53.2)
4–7 children	35 (5.4)
**Past experiences**	**n = 641 (%)**
Reported low mood or depressed in the last 12 months	334 (52.1)
Reported ever been verbally threatened, humiliated, repressed, frightened or made to feel worthless or unwanted	206 (32.1)
Reported ever physically abused in their lives	120 (18.7)
Reported ever been raped (forced to have sex against their will)	14 (2.2)
**Childbirth experience**	**n = 641 (%)**
Came directly to facility to give birth	497 (77.4)
Had previous delivery in current facility before	165 (25.8)
Reported complications during childbirth	403 (62.8)
Had cesarean section	100 (15.6)
Reported manual extraction of placenta	93 (14.5)
Baby died	39 (6.1)
**Satisfaction and quality of care**	**n = 639 (%)**
Satisfied with current delivery services	560 (87.6)
**Perceived quality of care received**	**n = 640 (%)**
Excellent	338 (52.8)
Good	224 (35.0)
Fair	78 (12.2)
**Time of delivery**	**n = 641 (%)**
Day	368 (57.4)
Night	273 (42.6)
**Type of sector**	**n = 641 (%)**
Public	583 (90.5)
Private	58 (9.1)

*in cases where the denominator is less than 641, there were missing data as a result of non-response which is not included in the analysis.

Over three quarters of the women reported going directly to the facility to give birth, and one quarter had delivered in the same facility previously. Fifteen percent had a cesarean section (n = 100), and 62.8% (n = 403) reported some sort of complications connected with their recent birth. Six percent (n = 39) of women reported the deaths of their recently delivered infants. The majority (87.6%, n = 560) of women expressed both satisfaction and perceived excellent/good quality of care. Forty-three percent (n = 273) of the deliveries occurred at night, and most (90%, n = 583) were in the public sector.

### Reported prevalence of disrespectful and abusive care during childbirth

Table 3 describes elements of D&A that women reported as experiencing during their facility stay. Self-reported prevalence of any D&A by postnatal women was 20% (n = 129). This was defined as any feeling of disrespect or humiliation during the childbirth experience. For responses to direct questions on different manifestations of D&A: 8.5% (n = 55) of women reported non-confidential care; 18% (n = 115) reported non-dignified care; and 14.3% (n = 92) reported neglect or abandonment. Non-consensual care was reported in 4.3% (n = 28) of cases. 4.2% (n = 27) of women reported physical abuse; 8.1% (n = 52) of women reported detainment for non-payment of fees, while demand for unofficial payment was reported at just less than one percent.

**Table 3:** Prevalence of reported disrespect & abuse during childbirth.

**Reported prevalence of D&A**	**n = 641 (%)**
Any treatment that made you feel humiliated or disrespect	129 (20.1)
	**n = 641**
**Non confidential care**	**55 (8.5)**
Treated in a way that violated privacy	47 (7.4)
Treated in a way that violated confidentiality	25 (3.9)
	**n = 639**
**Non-dignified care**	**115 (18.0)**
Provider talked or used a facial expression that made you feel uncomfortable	115 (18.0)
	**n = 641**
**Neglect/abandonment**	**92 (14.3)**
Left unattended by health workers when you needed help	81 (12.6)
Ignored regarding requests for pain relief	48 (7.5)
	**n = 638**
**Non-consented care**	**28 (4.3)**
Treatment given without permission	28 (4.3)
	**n = 637**
**Physical abuse**	**27 (4.2)**
Physical abuse (slapping pinched pushed, beaten, poked)	27 (4.2)
	**n = 641**
**Inappropriate demands for payment**	
Detention in facility for failure to pay	52 (8.1)
Request for a bribe for services	6 (0.9)

Note: women may report more than one occurrence of D&A

### Relationship between reported D&A and clients characteristics

A logistic regression analysis determined the association between various possible predictors of D&A and the categories of experience. Women between 20 and 29 years old were less likely to experience non-confidential care compared to these under 19 years of age; OR: [0.6 95% CI (0.36, 0.90); p = 0.017]. Women of higher parity, between one and three children, were three times more likely to be detained for lack of payment or five times more likely to be requested for a bribe compared to those who had just given birth to their first child; OR: [3.5 (2.2, 5.9); p<0.001] and OR: [4.5 (1.2, 17.4); p = 0.028] respectively. Clients were less likely to be detained for lack of payment or bribed if they were married; OR: [0.15 (0.07, 0.34); p<0.001] and OR; [0.19 (0.05, 0.72); p = 0.014] respectively; but women were twice more likely to be neglected if married compared to those that are single or never married: OR: [1.7 (0.9, 2.9); p = 0.052]. Clients with no support (such as a partner or companion) during delivery were less likely to experience inappropriate demands for payments or detention; OR: [0.49 (0.26, 0.95); p = 0.037] (Table 4).

**Table 4:** Relationship between reported disrespect and abuse during childbirth and client characteristics (OR 95%CI).

Characteristics	Any Disrespect	Physical Abuse	Non consented care	Non confidential care	Non dignified care	Detained: lack of payment	Request for bribe	Neglect
**Age: (reference group: below 19 years)**								
20–29 years	1.1 (0.61,1.8) p = 0.818	1.5 (0.70,3.3) p = 0.283	NA	0.6 (0.36, 0.90) p = 0.017	1.1 (0.55,2.3) p = 0.729	0.4 (0.12,1.4) p = 0.186	0.4 (0.092,1.7) p = 0.223	1.2 (0.4,3.6) p = 0.725
Over 35 years	NA	0.8 (0.03,17.4) p = 0.861	NA	0.29 (0.05,1.4) p = 0.132	NA	0.26 (0.03,1.8); p = 0.185	NA	NA
**Parity (reference group: no previous children)**								
Between 1–3 children	1.2 (0.59,2.3) p = 0.621	0.9 (0.46,1.9) p = 0.892	1.2 (0.35,4.5) p = 0.714	0.8 (0.56,1.1) p = 0.305	0.8 (0.38,1.8) p = 0.657	3.5 (2.2,5.9) p<0.001*	4.5 (1.2,17.4) p = 0.028*	1.3 (0.6,2.5) p = 0.442
Between 4–9 children	0.9 (0.13,6.8) p = 0.984	1.3 (0.20,8.3) p = 0.779	4.7* (1.1,20.7) p = 0.040	1.5 (0.87,2.8) p = 0.127	1.3 (0.4, 4.5) p = 0.609	12.4* (3.2,47.4) p<0.001	49.4* (8.6,27.9) p<0.001*	0.86 (0.2,3.7) p = 0.850
**SES: (reference group: top 80%)**								
Lowest 20%	0.96 (0.41,2.2); p = 0.938	NA	0.87 (0.2,3.9) p = 0.861	0.95 (0.37,2.4) p = 0.930	0.90 (0.35,2.2) p = 0.834	0.78 (0.35,1.7) p = 0.566	NA	0.61 (0.35,1.1) p = 0.089
**Education Attained (reference group: grade 1–4)**								
Grades 5–7	0.84 (0.51, 4.5) p = 0.857	NA	0.34 (0.03,3.2) p = 0.351	NA	1.7 (0.28,10.6) p = 0.557	NA	NA	1.2 (0.2,6.2) p = 0.809
Grades 8+	1.09 (0.26,4.5) p = 0.900	NA	0.25 (0.03,1.6) p = 0.157	NA	1.5 (0.27,9.1) p = 0.601	NA	NA	1.2 (0.2,6.2) p = 800
**Marital Status (reference group: Never Married & Separated)**								
Currently Married	0.66 (0.42,1.0) p = 0.067	0.90 (0.52,1.5) p = 0.709	2.2 (0.32,15.1) p = 0.420	1.9 (1.0,3.8) p = 0.048*	1.1 (0.43.2.5) p = 0.912	0.15 (0.07,0.34) p<0.001*	0.19 (0.05,0.72) p = 0.014*	1.7* (0.9,2.9) p = 0.052
**Family/Friend Present (reference group: no support)**								
Had support	0.59 (0.29,1.2) p = 0.163	2.3 (0.50,10.3) p = 0.288	0.61 (0.16,2.25) p = 0.465	0.89 (0.50, 1.6) p = 0.724	0.49 (0.26,0.95) p = 0.037*	0.53 (0.21, 1.35) p = 0.189	0.17 (0.02,1.2) p = 0.073	0.6 (0.36,1.0) p = 0.083

* Denotes a statistically significant finding with a p-value<0.05. For each category described in the table the reference groups are defined as indicated. NA- refers to cases where statistical analyses could not be performed

## Discussion

This study aimed to contextually define the types of disrespectful and abusive behaviors that manifest in Kenya, and measure their prevalence during the childbirth process. The overall prevalence of D&A reported among this study population of 641 postnatal women from 13 Kenyan facilities is 20%. Within the study facilities, women reported six main categories of D&A with prevalence ranging from 4 to 18% for different categories.

Both anecdotal and published work indicate that clients are often discriminated according to race, ethnicity, religion, age, socio-economic, and HIV status (12). This study tested the relationship between age, economic and marital status, parity, and support during childbirth, and education with any type of D&A. There are no statistical associations between different categories of reported D&A with client age, education, and socio-economic status. Lack of associations may be due to low levels of reporting associated with potential normalization of the different categories of D&A.

A protective effect seems to exist against occurrence of non-dignified care when a woman has a companion throughout labor and delivery [OR: 0.49 (0.26, 0.95); p = 0.037]. The main indicator for measuring non-dignified care in this context was the “provider talking or using a facial expression that makes clients feel uncomfortable”. This association appears logical, as providers will be cautious about how they speak to clients or relate to them when a companion of the client is present. Availability of support during childbirth is one area reported to have a positive effect for clients during the birthing process and is recommended in the national standards of care [16].

Clients with higher parities were more likely to be detained for lack of payment compared to women with no previous children; this was also the case women who were bribed for services. This observation is likely linked to better planning among primigravidae than women who have already had children. Women with higher parity (4–9 children) are also more likely to experience non-consensual care compared to those without prior children, which may be due to provider perception that multi-parous women already have previous birth experience. Another interesting association is evident between marital status and detention, bribery, and neglect. Married women were less likely to be detained for non-payment of user fees or bribed compared to those who are never married or separated. This observation may be associated with married women’s social networks as well as the fact married women may come from more stable households with access to funds.

The evidence presented here, is based on women’s self-reported experience of D&A during childbirth, and informs two key issues about D&A’s prevalence. First, women’s previous experiences of D&A at healthcare facilities, for childbirth or other visits, may “*normalize*” disrespectful or abusive care. Women expect such behavior and therefore do not think it is abnormal, illegal, or ethically wrong [12]. As a result of normalization, clients may not be able to distinguish between acceptable standards of care and those violating their patient and human rights. Second, women who have experienced disrespect, violence, or “*patriarchal privilege*” in their daily lives outside the health system may also be more likely to accept poor treatment within a facility. This is more likely in settings where the global estimate of gender-based violence (GBV) against women is high; recent research estimates GBV as ranging between 15 to 71% in many countries [17–21], with recent estimates from Africa indicating lifetime prevalence between 25 and 48% (i.e. 48% in Zambia, 47% in Kenya, 34% in Egypt, 30% in Uganda and 25% in South Africa) and annual prevalence ranging between 10 and 26% [22–24].

There are a few limitations to this study. Clients may have underreported the occurrences of D&A for two reasons. First, the interviews were held within the facility grounds and clients may have perceived that reporting D&A could jeopardize their future use of services at the same facility, especially for postnatal care. However the interviews were conducted in private conditions at facilities, where they were assured of confidentiality. Under- reporting may also be due to the fact that women have “*normalized*” some of the behaviors. Furthermore, this is one of the first studies to measure prevalence; we based much of the initial thinking on the landscape analysis developed by Bowser and Hill [12]. While this provided a detailed summary and excellent foundation, the framework itself has not yet been tested or validated. Nevertheless, nearly 20 percent of postnatal women reported some form of D&A, which indicates that serious D&A issues affect willingness to deliver in a health facility, which contributes to Kenya’s low SBA rate and ultimately reduces the likelihood of reaching MDG 5.

There is a growing body of qualitative literature describing disrespectful and abusive treatment during childbirth and poor quality of care experienced by women in a variety of settings [25–28], This paper is one of the first to describe the prevalence of D&A. These results have contributed to the design of a package of interventions in Kenya at policy, health facility and community level to ensure that women and providers understand that mistreatment is neither normal nor acceptable, and to create a culture of support, accountability and professionalism among policy makers, health managers and providers.

## Conclusion

One out of five women experienced feeling humiliated during labor and delivery. Six categories of D&A during childbirth in Kenya were reported. Women of higher parity were three times more likely to be detained for lack of payment, and five times more likely to be requested for a bribe compared to those who had just given birth to their first child. Understanding the prevalence of D&A is critical in developing interventions at national, health facility and community levels to address the drivers of D&A and to encourage clients’ future facility utilization. Further research is required to understand the extent of D&A in other regions.
